# Power monitoring in a feedforward photonic network using two output detectors

**DOI:** 10.1515/nanoph-2022-0527

**Published:** 2023-01-04

**Authors:** Sunil Pai, Carson Valdez, Taewon Park, Maziyar Milanizadeh, Francesco Morichetti, Andrea Melloni, Shanhui Fan, Olav Solgaard, David A. B. Miller

**Affiliations:** PsiQuantum, Formerly Stanford University, Palo Alto, CA, USA; Politecnico di Milano, Milan, Italy; Stanford University, Electrical Engineering, Stanford, CA, USA

**Keywords:** calibration, noninvasive power monitoring, perturbative measurement, photonic mesh, photonic neural networks, silicon photonics

## Abstract

Programmable feedforward photonic meshes of Mach–Zehnder interferometers are computational optical circuits that have many classical and quantum computing applications including machine learning, sensing, and telecommunications. Such devices can form the basis of energy-efficient photonic neural networks, which solve complex tasks using photonics-accelerated matrix multiplication on a chip, and which may require calibration and training mechanisms. Such training can benefit from internal optical power monitoring and physical gradient measurement for optimizing controllable phase shifts to maximize some task merit function. Here, we design and experimentally verify a new architecture capable of power monitoring any waveguide segment in a feedforward photonic circuit. Our scheme is experimentally realized by modulating phase shifters in a 6 × 6 triangular mesh silicon photonic chip, which can non-invasively (i.e., without any internal “power taps”) resolve optical powers in a 3 × 3 triangular mesh based on response measurements in only two output detectors. We measure roughly 3% average error over 1000 trials in the presence of systematic manufacturing and environmental drift errors and verify scalability of our procedure to more modes via simulation.

## 1 Introduction

Optical neural networks (ONNs) have long been proposed as candidates for fast and energy efficient machine learning and signal processing [1–4]. Recently, integrated photonic mesh networks of Mach–Zehnder interferometers (MZIs) [5, 6] have been shown to implement alternating unitary linear operators [7] and nonlinear optical layers to form optical neural networks. In such networks, the input and output data are complex numbers physically represented by the amplitude and phase of modes propagating through single-mode waveguides and networks of MZIs that interfere with these modes to ultimately form the physical optical transformation representing a unitary operator *U*. In other words, an *N*-port photonic mesh assumes an ideal input vector ***x*** of complex amplitudes in the input waveguides and a corresponding output vector ***y*** of complex amplitudes in the output waveguides related by the matrix-vector product ***y*** = *U* ***x***.

Cascading these linear optical devices with optical nonlinearities results in an all-optical neural network processor [3, 8, 9] that can potentially solve machine learning problems entirely in the optical domain. Crucially, these photonic meshes can be mass-manufactured in CMOS foundry photonics processes using silicon or silicon nitride waveguides and programmable phase shifters (electro-optic [10], thermo-optic [11], microelectromechanical [12], and phase-change [13]). Despite these obvious attractive properties, calibration and control of large multilayer programmable photonic circuits has previously proven a challenge, limiting many commercially viable applications such as photonic deep learning and blockchain technologies that require low systematic error [14, 15].

**Figure 1:**
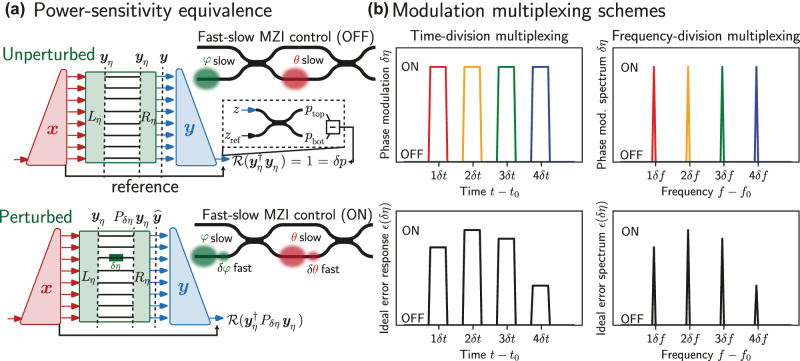
Power monitoring scheme: (a) The architecture of phase modulation sensitivity analysis is constructed from three subunits: the generator (red) to generate the signal, mesh (green) to transform the signal, and analyzer to compute signal transform monitor function via vector product (blue). Regardless of the actual input/output behavior (assuming no loss), modulation compares the response where all phase shifts are unperturbed and correctly set (OFF) to the response where to a given phase shift is perturbed (ON). In the ON state for a given phase shifter, subtracting the bottom waveguide from top waveguide gives the error modulation signal *ɛ*(*δη*(*t*)) from which the powers may be extracted as in Eq. (3). (b) Assuming some fixed *U*, ***x***, different multiplexing formats (top) result in different *ɛ*(*δη*(*t*)) (bottom, black) but ultimately yield the same response after demultiplexing. Whether the multiplexing is done in time or in frequency, either technique can isolate small-signal responses in the monitor function for the various different phase shifts (indicated by color) given necessary constraints (e.g., all frequencies must lie with an “octave,” or between *f*_0_ and 2*f*_0_, to avoid spurious readings during demodulation).

Accurate calibration and programming of feedforward photonic circuits benefits from non-invasive monitoring of intermediate powers as light propagates through the chip [16, 17]. The chip itself consists of a connected network of 2 × 2 MZIs (see Figure 1(a)) each implementing the transmission matrix *T* between its pairs of inputs and outputs

(1) $$T\left(\theta ,\phi \right)=ie^{i\frac{\theta }{2}}\left[\begin{array}{cc} -\sin\frac{\theta }{2} & \cos\frac{\theta }{2}\\ \cos\frac{\theta }{2} & \sin\frac{\theta }{2} \end{array}\right]\left[\begin{array}{cc} 1 & 0\\ 0 & {\text{e}}^{\text{i}\phi } \end{array}\right].$$

This device matrix is parameterized by internal phase shift *θ* and external phase shift *ϕ* which can be programmed by an external voltage. For instance, a thermal phase shifter operates by locally heating silicon material, which increases the mode effective index and, due to the corresponding increase in the phase delay through the phase shifter, causes light to interfere differently. As we will show in this work, such phase shifting elements can be simultaneously used as “power monitors,” for measuring the power propagating through the individual elements.

Currently, such power monitoring measurements are instead typically realized by either some power tap to a conventional photodetector, which necessarily introduces some additional loss in the propagation, or in-line devices based on capacitive coupling of existing photoconduction in additional lengths of silicon waveguides, which both introduces loss and increases the system length [17]. Here, we propose a more general and compact approach for monitoring based on systematically modulating individual phase shifters in the device using equivalent time- or frequency-multiplexed measurements, with an experimental demonstration of the time-multiplexed approach.

## 2 Methods

In our scheme, to perform direct power measurements, we modulate a phase shifter, and use balanced detection to compare the resulting modulation in output powers from the mesh on a pair of photodetectors as shown in Figure 1(a). The power through the given phase shift in the photonic mesh can be measured using the resulting modulation amplitude in the signal difference between these two photodetectors. With this general goal in mind, we now proceed to explain the overall procedure for power monitoring in the photonic mesh while proving our measurement technique mathematically.

We can represent the *P* phase shift parameters of the device implementing *U* (internal phase shifts ***θ*** and external phase shifts ***ϕ***) by a vector ***η*** = [***θ***, ***ϕ***] whose elements are the *θ* and *ϕ* phase shift values in all the various MZIs in the device. We now assume that a single phase shifter *η* is perturbed to yield *η* + *δη*, and the “perturbed vector” $\hat{\eta }=\left(\ldots ,\eta +\delta \eta ,\ldots \right)$ . So, for the same vector ***x*** of inputs, the set of output amplitudes is now $\hat{y}$ corresponding to the “perturbed device” $U\left(\hat{\eta }\right)$ . For convenience, we assume that the overall power (including power in the mesh and the reference arm) is 1 and that ‖ ***x*** ‖ = ‖ ***y*** ‖ = 1 due to the assumption of a lossless device implementing unitary *U*. The “monitor function” to measure the effect of the perturbation on the overall circuit is a root mean square function comparing the perturbed response $\hat{y}$ to the unperturbed response ***y***:

(2) $$\varepsilon \left(\eta \right)=\|y-\hat{y}\|=\|Ux-U\left(\hat{\eta }\right)x\|=\sqrt{2\left(1-R\left(y^{†}\hat{y}\right)\right)},$$

where R indicates real part.

**Figure 2:**
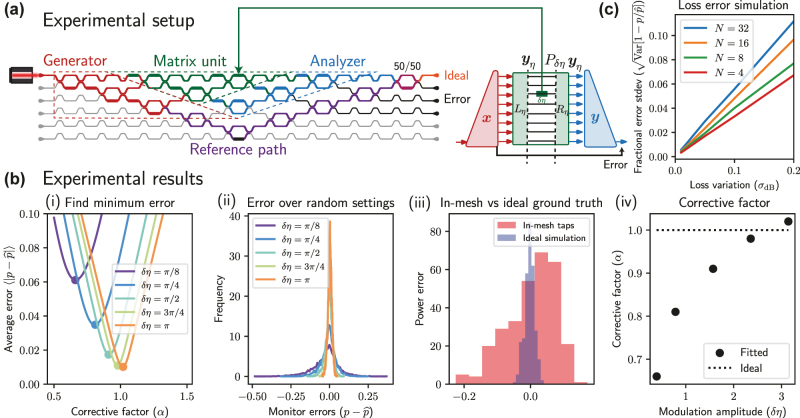
Power monitoring experiment and error scaling: our experiment is run using the setup in Refs. [14, 15]. (a) The experimental setup sends half the light to the “matrix unit” (green) which is the actual photonic mesh, with generator (red) and analyzer (blue) set to the desired input/output behavior. The rest of the light is sent into a reference channel (purple) which interferes at a 50/50 coupler (maroon), allowing for the real part to be measured at the top port (ideal). (b) Over 1000 trials, we monitor powers through six phase shifts given random 3 × 3 unitaries *U* and inputs ***x*** and compare expected *p* with measured $\hat{p}$ for different modulation amplitudes *δη*. (i) We measure an optimized corrective factor *α*, achieving an average error of 0.01 (roughly 3%) for *δη* = *π*. We apply this factor to measure (ii) a histogram of errors over random settings for various *δη* (in radians). (iii) We compare errors with respect to in-mesh and ideal simulation ground truths for 50 random settings and find much larger errors using in-mesh taps as ground truth (justifying the use of ideal simulation as ground truth) given *δη* = 2. (iv) Finally we show some deviation of the fitted corrective *α* from the ideal corrective factor α* = 1 especially at smaller *δη*, possibly caused by nonideal extinction ratio at the output (roughly 25 dB). (c) Using simphox [20], we analyze the scalability of our technique in the presence of loss variation, finding that the percent (fractional) standard deviation in error is on the order of the dB standard deviation *σ*_dB_ in losses across the circuit at scales up to *N* = 32, suggesting scalability of our technique.

To measure the monitor function of Eq. (2) physically, a convenient function for power monitoring as we show later, we propose the architecture of Figure 1(a) and program the specific circuit satisfying the properties of Figure 1(a) on 6 × 6 photonic mesh. The key insight is, as defined in Refs. [18, 19], to use a vector “generator” – a “diagonal line” (or more generally, in graphs theory terms, a binary tree) of 2 × 2 blocks to set inputs ***x*** (sent into the main feedforward mesh implementing *U*) and a similar binary tree “analyzer” to perform inner products with the original desired vector ***y***; with no monitor function perturbations introduced by changing any phase shifter values, the output from that analyzer would be an ideal value of 1. The analyzer output is also connected via a 50/50 directional coupler to a reference channel which provides a phase reference as shown as the dashed box in Figure 1(a). (In practice, we have implemented this 50/50 coupler using a final MZI on the right, as shown in Figure 2(a)). To the outputs of this coupler, we connect detectors to ultimately perform power monitoring in the circuit assuming that the reference channel and the analyzer output waveguide have the same optical power. Overall “balanced loss” in the device implementing *U* can be accounted for by allocating less light in the reference path at the input to equal the light exiting the output of the analyzer.

We write the reference field (mode amplitude and phase) into the 50/50 coupler as *z*_ref_. The output field phasor from the analyzer into this coupler is the inner product implemented by the analyzer $z≔y^{†}\hat{y}/\sqrt{2}$ . This presumes a power of 1/2 in the reference arm and 1/2 in the mesh (i.e., $|z|=|z_{\text{ref}}|=1/\sqrt{2}$ ). If we choose the phase of the reference arm such that $z_{\text{ref}}=-i/\sqrt{2}$ (i.e., a phase of −*π*/2), the difference in the powers of the output coupler *δp* ≔ *p*_top_ − *p*_bot_ can be related to the monitor function *ɛ*(***η***) as follows (also diagrammatically represented in Figure 1(a)):

(3) $$\begin{array}{cc} \left[\begin{array}{c} q_{\text{top}}\\ q_{\text{bot}} \end{array}\right] & =\frac{1}{\sqrt{2}}\left[\begin{array}{cc} 1 & i\\ i & 1 \end{array}\right]\left[\begin{array}{c} z\\ z_{\text{ref}} \end{array}\right]=\frac{1}{\sqrt{2}}\left[\begin{array}{c} 1/\sqrt{2}+z\\ -i/\sqrt{2}+iz \end{array}\right]\\ p_{\text{top}} & =|q_{\text{top}}{|}^{2}=\frac{1/2+|z{|}^{2}+R\left(y^{†}\hat{y}\right)}{2}\\ p_{\text{bot}} & =|q_{\text{bot}}{|}^{2}=\frac{1/2+|z{|}^{2}-R\left(y^{†}\hat{y}\right)}{2}\\ \delta p & =p_{\text{top}}-p_{\text{bot}}=R\left(y^{†}\hat{y}\right)=1-\varepsilon^{2}/2 \end{array}$$

The *δp* quantity could be measured directly using balanced photodetector circuits that subtract signals between pairs of output photodetectors, though we perform this subtraction operation digitally in this work.

Based on the monitor function *ɛ*(***η***) of Eq. (2), we seek to prove that the monitoring measurement of Eq. (3) and inset of Figure 1(a) is exactly the power in the phase shifter *η*(*p*_*η*_) within a lossless photonic circuit implementing *U*. We define device operators such that $\hat{U}≔U\left(\hat{\eta }\right)=R_{\eta }P_{\delta \eta }L_{\eta }$ and *U* ≔ *U*(***η***) = *R*_*η*_ *L*_*η*_, where *L*_*η*_, *R*_*η*_ represent operators before and after (to the left and right) of the phase shift perturbation *δη* in a given device and *P*_*η*_ is the operator for some applied *η* phase shift, i.e., a e^i*η*^ phase shift is applied to any given single waveguide mode (e.g., mode *m*) of the system. We also define the mode vector of phasor quantities at the phase shifter to be ***y***_*η*_ as indicated in Figure 1(a), with elements denoted as *y*_*η*,*m*_, where *m* = *m*_*η*_ indicates the waveguide where is a phase perturbation *δη* applied. Note that $p_{\eta }=|y_{\eta ,m_{\eta }}{|}^{2}$ by this definition.

Given the above definitions, monitor function *ɛ* ^2^(*δη*) is now defined as follows:
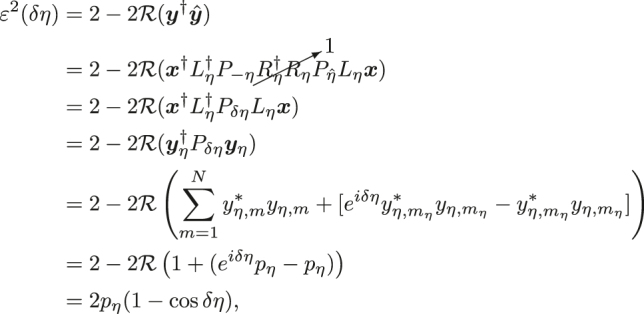
 (4) where in the final step of Eq. (4), we can either choose small *δη* for which *δη* ^2^ ≈ 2(1 − cos *δη*) or the optimal *δη* = *π*, which ensures the maximum signal amplitude 4*p*_*η*_. In the case of small *δη*, $\sqrt{p_{\eta }}\approx \varepsilon \left(\delta \eta \right)/\delta \eta$ , which is to say that the field amplitude at phase shifter *η* is equal to the monitor function *ɛ* divided by the phase shift modulation amplitude *δη*. Equivalently, we might consider $\sqrt{p_{\eta }}$ as a “sensitivity” because it is the proportionality constant relating the overall circuit monitor function to the change in the single phase shift. A more detailed analysis of this type of model using second-order Hessian perturbations is provided for binary tree feedforward circuits in Ref. [19]. In practice, as will be shown later, we will need to define a “corrective factor” *α* to relate *ɛ* ^2^(*δη*) to the measured power due to various non-idealities such as extinction of the output signal (which is related to circuit calibration error and component error), giving *ɛ* ^2^(*δη*)*α*.

The mechanism for measuring powers *p*_*η*_ may rely on either time-based or frequency-based multiplexing (Figure 1(b)) to measure *ɛ*(*δη*) for many phase shifters *η* at a single photodetector represented by the output of the dot product analyzer implementing the ideal ***y***. The protocol ultimately proceeds as follows given inputs ***x*** and device implementing *U*(***η***):

1. An arbitrary input mode vector ***x*** is generated at the input using a generator (red in Figure 1(a)).
2. The feedforward mesh transforms input modes ***x*** into output modes $\hat{y}=U\left(\eta \right)x$ (green in Figure 1(a)).
3. Using self-configuration, the analyzer unit guides the output $\hat{y}$ into a single waveguide (blue in Figure 1(a)).
4. The phase at the output of the ***y***-analyzer (effectively the “global” phase degree of freedom for ***y***) is adjusted so that interfering with the reference beam yields all the power in the top waveguide, i.e., $\delta p=R\left(y^{†}\hat{y}\right)=1$ , since when *δη* = 0 for all *η*, we have $y=\hat{y}$ (no perturbations).
5. Perturb the voltages ***v*** (containing *P* voltages *v*_*p*_ for *p* ≤ *P*) by some amplitude *v*_*a*_(*δη*) (relating voltage to phase amplitude) multiplexed in time or frequency as in Figure 1(b).
6. While applying the modulation, track the monitor function stage measuring *ɛ* using Eq. (3), a scalar objective comparing $\hat{y}$ and ***y*** as in Eq. (2), by subtracting the power in the top output from the power in the bottom output of the reference output 50/50 coupler as shown in Figure 1. For practical purposes (e.g., if high extinction ratio cannot be achieved), optimize the corrective global scaling factor *α* to minimize the error between measured and predicted powers.

## 3 Results and discussion

As shown in Figure 2(a), we have implemented a demonstration of our technique using a 6 × 6 mesh within which we are able to embed a 3 × 3 triangular mesh (green), 1 × 3 analyzer and generator (blue and red respectively), and a reference channel (purple). Power in all output waveguides are measured via fiber photodetectors coupled from output gratings of the chip, and we measure *δp* as in Eq. (3) by subtracting the power measured by the photodetectors in the top and bottom waveguides. We define two ground truth measurements for the expected intermediate powers *p*: (1) we simulate an ideal mesh and power propagating through the mesh (2) for a smaller set of measurements (50 samples), we measure (using an IR camera) intermediate optical powers in the mesh via grating taps placed next to each phase shifter to justify why (1) is an appropriate ground truth choice due to what is likely coupling errors in the grating taps (panel (iii) in Figure 2(b)). We compare this to the measured $\hat{p}$ using our technique. More details on the experimental system (e.g., calibration and input vector setting) are provided in Refs. [14, 15].

Due to simplicity, we use the time multiplexing scheme (effectively the analog equivalent to the finite-difference method) in this work to extract powers passing through the various different phase shifters. The protocol for this is fairly straightforward, which is to pulse each phase shifter in succession and read out the appropriate output response *δp* = 1 − *ɛ* ^2^/2 in the same sequence as each phase shifter is individually perturbed, achieved using synchronous updates and readouts.

Our main results, shown in Figure 2(b), indicate that with sufficiently high modulation amplitude (the optimal one at *δη* = *π*) we can achieve around 0.01 standard deviation in error modulation ( $\sqrt{\mathrm{Var}\left(p-\hat{p}\right)}$ , distributions shown in panel (ii)) and average error ( $\left\langle \left|p-\hat{p}\right|\right\rangle$ , shown in panel (i)), which is roughly 3% of the expected average fractional power in each waveguide (1/3, since the mesh supports 3 waveguide modes). We achieve an extinction ratio of 25 dB in our ideal output channel, a leakage of optical power which results in the need to determine corrective factors for smaller modulation amplitudes as shown in panel (iv). As indicated by panel (i), the error average and standard deviation decreases as the modulation amplitude approaches optimal *π* (or correspondingly, the signal-to-noise ratio) increases.

Note that when computing the sensitivity, both systematic and noise errors contribute as investigated in a previous study [14]). We find that systematic error (in the analyzer, generator, reference path, and feedforward mesh) tends to dominate in our chip from previous studies [14, 15], and we have reduced noise due to our use of fiber photodetectors instead of grating tap measurements at the output. Designing a low-loss and accurate input generator and output analyzer is critically important to mitigating this source of error in the future; robust “balanced binary tree” designs of such devices are discussed in Ref. [19].

Although we do not demonstrate frequency multiplexing experimentally, it is still worth discussing some of the constraints of the frequency-based scheme (shown in Figure 1(b)) to compare it with the time-based multiplexing scheme we use in this work. For the frequency modulation schemes, we first modulate each drive voltage *v*_*p*_ at frequency *ω*_*p*_, which in vectorized form is $\delta_{F}\left(t\right)=\sin\omega t$ where ***ω*** = (*ω*_1_, *ω*_2_, … *ω*_*P*_), similar to the harmonic orthogonal perturbation scheme as discussed in Ref. [21] originally aimed for model-free gradient measurements. We can parallelize our modulation across at least *P* elements in the device by applying a different *ω*_*p*_ over tunable voltage elements $v\in {\left[v_{\min},v_{\max}\right]}^{P}$ . The feedback control stage filters out variations in monitor function *ɛ* at different *ω*_*p*_ using a lock-in amplifier tuned to *ω*_*p*_, which is able to read out the various frequencies shown in Figure 1(b). Critically, this of course requires some *minimal integration time* to separate out the frequencies, which is on the order of the inverse of the frequency separation. To minimize second-order cross modulation effects, all *ω*_*p*_ are equally spaced apart within an octave near the modulation bandwidth Ω, i.e., *ω*_*p*_ ∈ [Ω/2, Ω] spaced *δf* = Ω/2*P* apart or $\omega_{p}=\frac{\Omega }{2}\left(1+\frac{p}{P}\right)$ . For thermal modulation [11], we can drive any voltage element *v*_*p*_ at a switching frequency limit of over Ω = 100 kHz, which can be raised to over Ω = 1 MHz for MEMS/NEMS modulation systems [12] and even as high as Ω = 1 GHz in electro-optic phase shifter systems (reaching a desired “fast” phase shifter regime as we discuss later). The limit of Ω/2*P* is on the same order as the switching time between various time bins in the time multiplexing scheme, which is actually takes 1/Ω time per phase shifter and a total time *P*/Ω for all phase shifters, actually twice as fast as this frequency binning scheme. However, the choice of frequency-based multiplexing might be motivated by any hardware configuration that favors frequency-based measurements over time binning, which can be explored in a future work. For the purposes of this paper, it suffices to simply show that our mathematical derivations are confirmed by experimental measurements.

Another practical consideration in our system is scalability to larger photonic circuits, which requires considering loss variation in the circuit because such error violates the assumptions in the proof of Eq. (4). Although larger circuits suffer from increasingly large errors due to loss variation, Figure 2(c) indicates that as long as the dB variation in loss is sufficiently low, our technique can scale to circuits with larger numbers of modes (e.g., fractional error for *N* = 32 is just twice as sensitive to loss variation compared to that of *N* = 4). Another consideration is the added footprint and error of the analyzer structure in the circuit into our procedure. Analyzer and generator circuits only require an total optical depth of 2 log  *N* (smaller than *N* depth for a universal mesh) and contain a total of 2*N* MZI nodes (smaller than *N(N - 1) / 2* nodes for a universal mesh). For the triangular mesh, as in our case, it is sufficient to use an *N* + 3-mode triangular mesh to track powers in an *N*-mode triangular mesh. Thus, the contribution to footprint (as well as the error contribution [19]) is negligible, particularly in the case that the monitored photonic mesh is used for general unitary operations.

Our power monitoring approach can be applied to the specific problem of *in situ* backpropagation in ONNs, which we have previously experimentally demonstrated to train neural networks deployed on photonic matrix accelerator chips [15]. Such a technique expands on the theoretical premise of Ref. [1] that first-order optimization of physical hardware to achieve some desired implementation is an experimental analogue of photonic inverse design optimization. Indeed, our proof and experimental demonstration of sensitivity-power monitoring equivalence can be used to implement the necessary measurements in our backpropagation technique in place of “invasive” grating taps used in Ref. [15] required in each waveguide segment of the device possibly with higher accuracy as found in Figure 2(b), panel (iii). Such a power-monitoring technique would be admittedly much slower and less energy efficient than using taps (due to the analyzer self-configuration and phase shift multiplexing), but the main benefit could be that it avoids needing additional electrical contacts for integrated photodetector taps.

To ultimately facilitate high-speed operation of power monitoring in ONNs, we propose a “fast-slow” scheme for efficient adaptive control and optimization of phase shifts. Fast phase shifters used for power monitoring maintain high switching bandwidth and occupy a small footprint assuming they do not achieve the full 0 → 2*π* range (Of course, this would require that the output signal-to-noise must be sufficiently high for such a “small” modulation amplitude). Next to these phase shifting elements are the slow phase shifters responsible for setting the actual photonic network weights that change infrequently, achieve the full 0 → 2*π* range, and occupy a larger footprint. This scheme is indicated in both on and off states via *δϕ*, *δθ* in Figure 1(a). Fast (e.g., electrooptic) phase shifters and slow phase shifters can operate using different physical mechanisms; for instance, use of barium titanate or lithium niobate Pockels fast phase shifters [10] next to thermal or MEMS slow phase shifters [11, 12] is one possibility. Though phase-modulated power monitoring (limited by phase shifter switching) is still slow compared to using an invasive technique limited by photodetector signal-to-noise ratio (SNR), the fast-slow phase shifter configuration can help to mitigate some of these concerns. Use of a high-frequency modulation for demultiplexing the monitor function signal response can also help because practical noise considerations (such as 1/*f* noise) are often much less of a concern at high modulation frequencies. This fast-slow scheme can be useful for a number of applications besides power monitoring, including training of optical neural networks according to stochastic schemes [8].

## 4 Conclusions

This paper highlights an important equivalence between circuit sensitivities to phase shifters and power flowing through those phase shifters. We have presented and experimentally tested a new architecture that is capable of monitoring powers flowing through any feedforward mesh [22] with applications towards backpropagation training of optical neural networks by tracking modulation in a balanced photodetector. This noninvasive power monitoring can be vitally important for calibration and optimization of various arbitrary photonic components and circuits, e.g., for photonic neural networks and photonic sensing circuits which may be useful for applications ranging from machine learning to telecommunications.
